# Effect of ultrasound on protein functionality

**DOI:** 10.1016/j.ultsonch.2021.105653

**Published:** 2021-06-24

**Authors:** Jing Su, Artur Cavaco-Paulo

**Affiliations:** a Jiangsu Engineering Technology Research Centre of Functional Textiles, Jiangnan University, 214122 Wuxi, China; b Key Laboratory of Eco-textiles, Jiangnan University, Ministry of Education, China; c International Joint Research Laboratory for Textile and Fiber Bioprocesses, Jiangnan University, 214122 Wuxi, China; d Center of Biological Engineering, University of Minho, Campus de Gualtar, 4710-057 Braga, Portugal

**Keywords:** Ultrasound, Protein functionality, Mass transfer, Enzymatic reactions

## Abstract

The review focus on the effect of ultrasound on protein functionality. The presence of transient ultrasonic mechanical waves induce various sonochemical and sonomechanical effects on a protein. Sonochemical effects include the breakage of chains and/or the modification of side groups of aminoacids. Sonomechanical modifications by enhanced molecular agitation, might lead to the transient or permanent modification of the 3D structure of the folded protein. Since the biological function of proteins depends on the maintenance of its 3D folded structure, both sonochemical and sonomechanical effects might affect its properties. A protein might maintain its 3D structure and functionality after minor sonochemical effects, however, the enhanced mass transfer by sonomechanical effects might expose internal hydrophobic residues of the protein, making protein unfolding to an irreversible denatured state. Ultrasound enhanced mass transport effects are unique pathways to change the 3D folded structure of proteins which lead to a new functionality of proteins as support shield materials during the formation microspheres. Enzymes are proteins and their reactions should be conducted in a reactor set-up where enzymes are protected from sonic waves to maximize their catalytic efficiency. In this review, focused examples on protein dispersions/emulsions and enzyme catalysis are given.

## Highlights

• Proteins can be modified by sonochemical and by sonomechanical means.
• Ultrasound enhanced transport effects are unique ways to change the 3D folded structure of proteins.
• Ultrasound effects enhances enzymatic reactions.

## Graphical abstract

**Figure:**
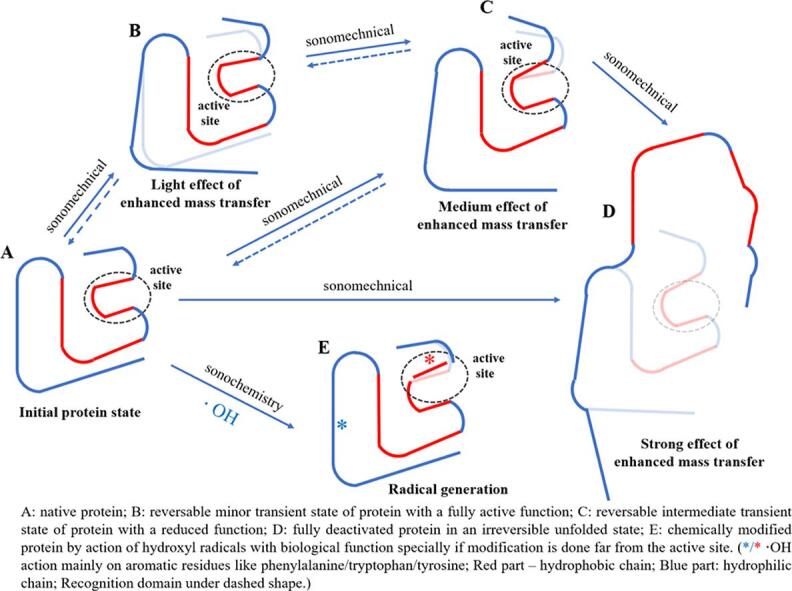

## 1 Introduction

Ultrasound technology is a non-isothermal and non-toxic processing technology which has been explored in various chemical and biochemical areas [1], [2], [3]. The main effect of ultrasound on liquid systems are cavitation, which is considered as the most significant effect dating from ultrasound process, generating hot spots, efficient mass transfer, highly reactive free radicals, and other phenomena, leading to an intensification of physical and chemical operations. Ultrasound has been applied in various processes due to its safety feature, namely the mixture/separation/extraction of liquid foods, the activation/inactivation of enzymes, the emulsification and functionality modification of proteins, etc [4], [5], [6].

In food, pharmaceutical and textile technologies, proteins can be modified using ultrasound process by altering their structural and physiological properties. For instance, Hu et al. studied the effect of ultrasound on the soy protein isolate (SPI) dispersions and found that ultrasonic treatment could improve the solubility and fluid character of SPI dispersions and benefit the subsequent operations [5], [7], [8]. Some other studies have investigated the use of ultrasound on the hydrolysis or the conjugation of the proteins. The research from Tian [5] demonstrated a significant effect of ultrasound on the conformational and physicochemical properties, promoting the degradation of peptide aggregates by breaking the non-covalent interactions. While, the findings from Ma [9] showed an enhancement for the conjugation between SPI and citrus pectin or apple pectin, and the results from Hui approved the intensification of the covalent conjugation between egg white protein and tea polyphenols [10].

Sonication induces cavitation which generates transient bubbles that when collapses, produce vast reactive free radicals which contribute to the processes [7], [11]. Rahman [7] proposed that most of the modifications in protein are derived from free radicals especially hydroxyl radicals. These free radicals could induce the oxidation of the amino-acid side groups in the protein chains. The most susceptible side chains are the aromatic groups in phenylalanine, tyrosine and tryptophan residues. But no systematic studies exist on the literature either reporting chemical modification or breakage of protein chains. Cysteine are claimed to be modified during the fabrication of microspheres [12]. But there are no experimental evidence in literature about inter or intra-protein crosslinking. One can expect that the modification of a small amount in amino acid side chains would result in minor changes in secondary and tertiary structures. Proteins with a smaller amount of aromatic residues would therefore not significantly change their biological function.

The biological functions of most globular proteins are associated with a molecular recognition process. This process is determined by the 3D structure at the protein surface where a molecule can specifically “seat-on”, for example, the recognition of the substrate in enzyme catalyzed reactions. When proteins work biologically, they must therefore keep the 3D folded structure and tend to be less tolerant to external changes like protein unfolding, which explains why proteins could be slightly modified at their primary structure (break of amino acid chain or radical reactions) without the activity loss by sonochemistry. However, enhanced mass transfer effects of ultrasound may lead to the protein transient unfolding with a considerable loss of activity like in enzymes, and ultrasound has the ability to change the 3D structure of a protein like albumin and adapt their structure to emulsify oils yielding stable structure up to years [13], [14], [15], [16], [17], [18].

The present review aims to give a focus overview on the use of ultrasound to modify the functions of protein or the enzymatic reactions applied in different areas. Focused examples from protein dispersions/emulsions, protein changes and enzyme catalysis reactions are given. The role of ultrasound on the above applications are evaluated. The strengths and weakness of the ultrasound process are pointed out, and the key points for the further applications in food, pharmaceutical and textile industries.

## 2 Ultrasound: a tool for the formation and stabilization of protein emulsions and dispersions

As a high energy methodology, ultrasound has been used in the formation of protein emulsions and dispersions with uniform and small droplet size for medical, pharmaceutical and cosmetic applications [19], [20]. Generally, protein emulsions in micro- and nano- scale could be obtained by low- and high-energy intensification [21]. For low-energy intensification, the emulsions are formed normally by phase inversion and spontaneous emulsification methods. These approaches are limited to large scale application due to the restricted selection of surfactants and their combination. High-energy intensification is applicable since the emulsions are formed using mechanical equipment including colloid mills, high-pressure homogenizers, and ultrasonic devices. When using various strategies, it is of crucial importance to evaluate the properties of emulsions produced including appearance, size, stability, rheology and others [22].

Martins and co-workers developed a new methodology for enzyme stabilization under ultrasonic fields for bleaching process, and the micro-emulsions made of laccase and bovine serum albumin (BSA) were prepared [16]. The formed proteinaceous micro-emulsions are packed sphere with a minimal contact with water, and laccases in this form show a relatively good activity towards extreme conditions like temperature, pH, and high shear forces. Compared with the free laccase, the modified enzyme shows a double half-life time, thus increasing the subsequent step of bleaching.

**Fig. 1:**
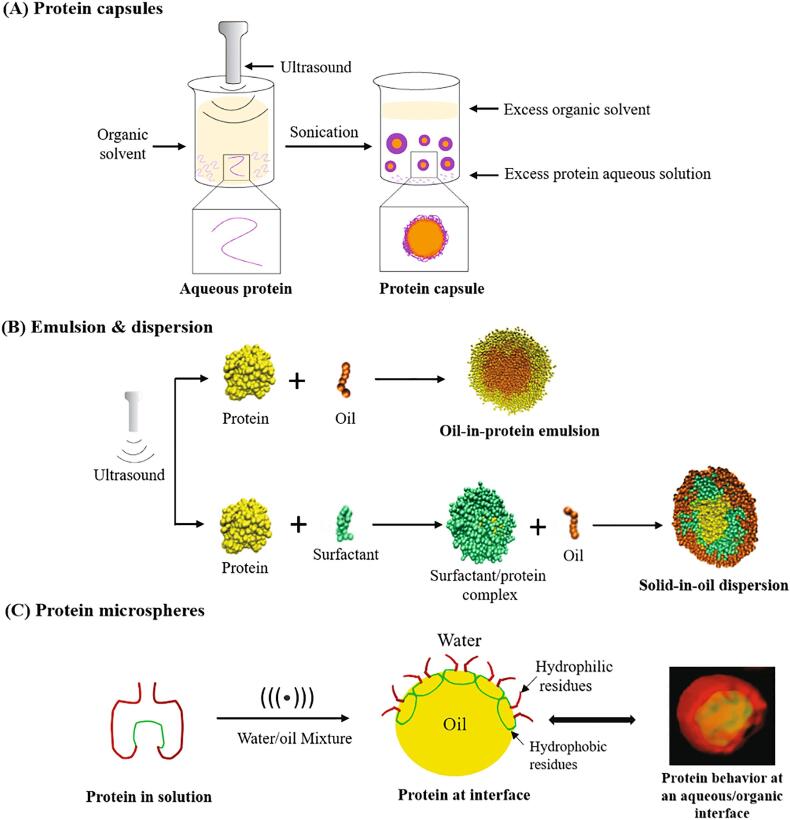
Illustration of Protein capsules, protein nanoemulsions/microemulsions and dispersions using ultrasonic emulsification method (adapted with permission from references [15], [20], [21]).

They also focus on the study of protein emulsions and formulations which could be used to form, stabilize, and deliver active components in the drug delivery system. They previously investigated the formation of the protein emulsion-based formulations like protein capsules and others using ultrasonic process (Fig. 1) [17], [19]. Ultrasound is capable to generate enough energy for emulsification, which favors the auto-aggregation of the proteins (Fig. 1A). This phenomenon may be explained by the formation of high turbulence that creates molecular agitation.

In general, emulsions in nano- or micro- scale are not stable which could turn into two or more phases. Their stability depends on the size of emulsion droplets, particle size distribution, emulsifier type, concentration, surface tension and others, of which the size of formed droplets influence greatly the properties of dispersed emulsion(Fig. 1B) [12]. The protein microspheres are formed due to high mechanical energy, inducing of protein re-conformation at oil–water interface (Fig. 1C) [18]. First studies conducted by Suslick [12] claim that the formation of microspheres were due to presence cysteine, but recent work [18] showed that microsphere can be formed with peptides without cysteine supporting the view that protein re-conformation at oil–water interface would be the most plausible explanation. Studies show that suitable sonication amplitude, sonication time and process temperature could generate the comprehensive interaction between them, contributing to the formation and stabilization of the emulsions and dispersions [21]. In addition, the stabilization of droplets against coalescence also play an important role for the stability, and a variety of emulsifiers/stabilizers such as surfactants and proteins can be introduced to the system to prevent the agglomeration and coalescence of the droplets formed [23]. The adsorption of emulsifiers like protein show a great potential to produce emulsion-based products as drug delivery systems for intravenous therapeutic application.

## 3 Ultrasound: an energy for conformational changes of protein structure

As mentioned, ultrasound has been employed as a method of enzyme activation and inactivation depending on appropriate or inappropriate conditions applied [24]. It is difficult to clarify the specific enzyme activation or inactivation mechanism since several chemical and physical effects occurred at once. However, it is known that the changes of enzyme activity depend mainly on the amino acid composition and protein conformation [15], [25].

The use of laccase as a promising catalyst has rapidly attracted the attention in various researches using ultrasound process [11], [26]. Laccases (EC 1.10.3.2) are a family of multi-copper containing oxidoreductases which could oxidize a range of substrates like phenolic, aromatic and some other compounds using only air oxygen under mild conditions [27]. Laccase-catalyzed reactions have been extensively studied from researchers due to its potential multi-functional reaction products applied in textile, medical and cosmetics areas [28], [29]. No detailed information exists about the effect of ultrasound on the structure of laccase enzyme proteins in previous studies [30], [31].

To explore, we evaluated the role of ultrasound on the polymerization of catechol using laccase as catalyst in previous studies [33]. When ultrasound was applied, the effects of the sonication are mainly high levels of transport which increase the mass transfer and create more propagation sites, enhancing the interaction between enzymes and reactants in aqueous ultrasonic system [34]. The data obtained show an intensification effect for the reaction with higher conversion yield and longer polymers as expected. The higher mass transport generated by ultrasound is the main feature responsible for a higher substrate accessibility to the enzyme which contributed to produce longer polymers.

**Fig. 2:**
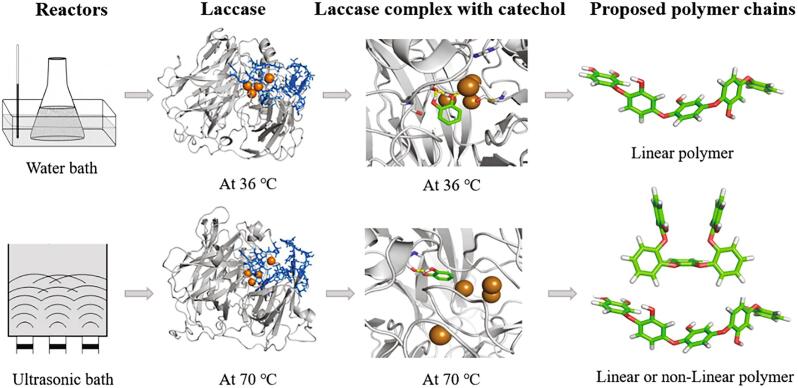
Protein conformation structures and proposed polymer structures under different processing conditions. Left side(lane 1): water bath (low-energy) and ultrasonic bath (high-energy); at the center (lane 2,3): the middle structures of laccase at 36 °C and 70 °C represented in cartoon, active site and cavities for catechol access to the T1 copper site are highlighted using blue amino acid side chains; laccase (grey) and copper atoms (orange), catechol in green; right side (lane 4): proposed structures of poly(catechol) obtained by quantum calculation, at B3LYP/6-311++G(d,p) level (adapted with permission from references [32], [33]). (For interpretation of the references to colour in this figure legend, the reader is referred to the web version of this article.)

Furthermore, the molecular dynamic simulation studies revealed the changes of active site of middle structure of laccase under ultrasound process (as shown in Fig. 2) [32], [33]. The cavitation phenomena generate an increase of local temperature as main form, thus simulated annealing method was performed to study the behavior of laccase under heating. Our simulation results showed that ultrasound treatment lead to a more open enzyme active site resulted from favorable conformational changes without altering its structural integrity. This change may play a crucial role in the catalytic efficiency, stability, and selectivity, which generate products with different structures or products at different stages. It is worth noting that when ultrasound applied, the half-life time of the enzyme could be reduced, however, appropriate frequency and power parameters of ultrasound could speed up the enzymatic reactions before protein inactivation [33], in this case the activity loss will not restrict the final product produced.

## 4 Ultrasound: as medium for intensification of enzymatic process

Recently enzymatic synthesis of polyester offers a new approach to these popular biodegradable polymers refraining from harsh chemical processes, which have been applied in textile, biomedical and other areas. Lipases are well known enzymes for this purpose which could catalyse esterification and transesterification reactions in the presence of aqueous medium [35], organic solvents and ionic liquids [36], or in solvent-free conditions [37]. A novel solvent-free enzymatic catalysis system provides desired reaction environments and does not require further purification process, during which reactants are intermiscible while enzymes are in a different phase, has been proved as potential green alternatives.

**Fig. 3:**
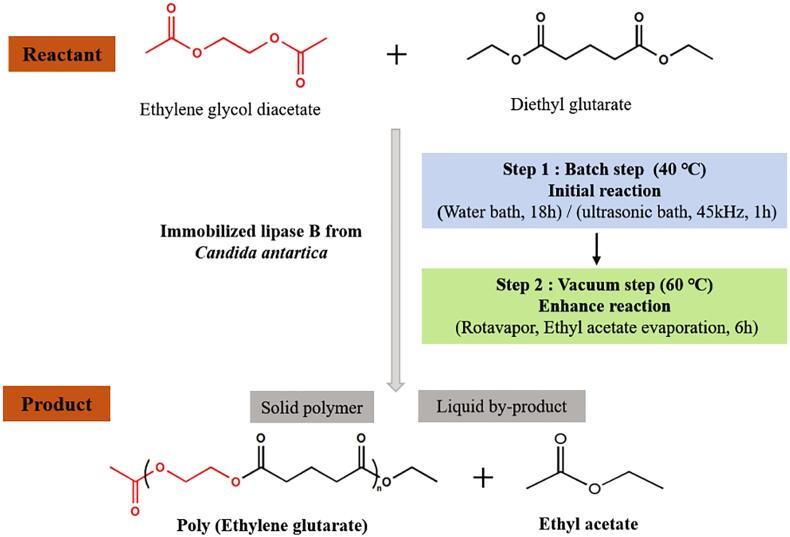
The route of lipase-catalyzed synthesis of poly(ethylene-glutarate) using a solvent-free system (adapted with permission from references [38]).

In this study our researchers investigated lipase catalysed synthesis of aliphatic polyester from diethyl glutarate and ethylene glycol diacetate in a solvent-free reaction [38]. To overcome the poor stability of lipase, an immobilized lipase was introduced to our process for better activity and stability [39]. However, when immobilized lipase and solvent-free system are both applied, the reactants exbibit a relatively slow rate of reaction due to limited reactants diffusion and low mass transfer. Under the circumstances ultrasound technique is expected to address this issue. A normal water bath and an ultrasonic bath were both employed for comparison, and the set of reactions are explained in Fig. 3.

The initial reaction kinetics study using different lipase concentrations was performed to verify the role of ultrasound treatment during the reaction. The data obtained support the intensification of reaction with the assistance of ultrasound and this enhancing effect are more significant when lower lipase concentration applied. For further confirmation, the reactions were proceeded for different reaction time using both water bath and ultrasonic bath, then an obvious shorten of reaction time of ultrasound process occurred (1 h of ultrasonic bath *vs* 18 h of water bath), which also testify the intensification effect.

**Fig. 4:**
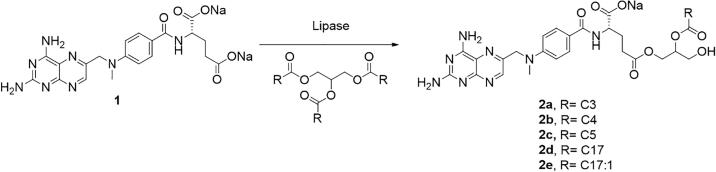
Reaction scheme of methotrexate-acylglycerols conjugates. Compound 2a,2b and 2c were produced in ultrasonic bath with immobilized lipase using glycerol tributyrate, glycerol trivalerate and glycerol trihexanoate, separately; Compound 2d and 2e were produced in ultrasonic bath with liquid lipase using glycerol tristearate and triolein, separately (with permission from reference [36]).

Moreover, our researchers conducted another set of studies to synthesis new methotrexate-acylglycerols conjugates using ultrasound-assisted lipase catalyzed system. For methotrexate-acylglycerols conjugates, both immobilized CALB and liquid lipase from *thermomyces lanuginosus* were used, and triacylglycerols with different carbon length (between C3 to C17) were selected. The reaction schemes for the conjugation of MTX with triacylglycerols are proposed in Fig. 4.

The conversion yields of the reactions by both enzymes were shown in Table 1. One could see that for short triacylglycerols used, immobilized lipase under ultrasonic bath display a better activity and lead to a higher yield due to the disappearance of the biphasic system and the better mixing of reactants. However, for longer triacylglycerols, it does not present any activity, corroborating with the previous study that immobilized lipase did not hydrolyse triolein in organic medium, and this behavior could attribute to the poor mobility of enzyme and the big size of triacylglycerols [40].Considering this, a liquid lipase was applied without any solvent, and we could observe the formation of methotrexate-acylglycerol conjugates using longer triacylglycerols, which could be explained by high mobility and activity of this enzyme and its accessibility to longer substrates. Although different behaviors observed upon different triacylglycerols, both enzymes under ultrasonic process demonstrate a better catalytic action than in normal water bath.

**Table 1:** Reaction conditions and conversion yield obtained from lipase-assisted conjugation of methotrexate-acylglycerols (data cited with permission from reference [36]).

	Triacylglycerol	Carbon Chain Length	Reaction conditions	Yield (%)*	
				Immobilized CALB	Liquid lipase *TL*
1	Glycerol tributyrate	C3	Water bath#1	_^a^	_^a^
2			Ultrasonic bath#2	61.4 ± 4.0	_^a^
3	Glycerol Trivalerate	C4	Water bath#1	_^a^	_^a^
4			Ultrasonic bath#2	64.1 ± 0.4	_^a^
5	Glycerol Hexanoate	C5	Water bath#1	45.8 ± 1.7	_^a^
6			Ultrasonic bath#2	58.0 ± 7.6	_^a^
7	Triolein	C17:1	Water bath#1	_^b^	_^a^
8			Ultrasonic bath#2	_^b^	62.6 ± 4.3
9	Glycerol Tristearate	C17	Water bath#1	_^b^	_^b^
10			Ultrasonic bath#2	_^b^	63.7 ± 6.4

#1 Water bath: (40 °C, 30 min).

#2 Ultrasonic bath: (5 min ON/5 min OFF, 30 min).

* Yield: the yield was calculated from the initial number of moles and the moles of products.

a Vestigial amount of conjugate product was detected by ^1^H NMR.

b No reaction occurred.

Apart from enzymatic synthesis of polyester, the enzymatic hydrolysis of polyester shows an enormous potential for functionalization and recycling applications. The first study on hydrolysis of aromatic/aliphatic polyesters by Hsieh could trace back to the 1990ies [41]. Ever since, a variety of enzyme from different organisms have been described to hydrolyse polyester, and enzymes belonging to the class of cutinases display an excellent performance. However, the efficiency of synthetized polyester is relatively lower compared with natural polyesters due to enzyme specific, thus different approaches like genetic engineering and ultrasound treatment have been exploited to enhance enzymatic activity. Pellis [42] conducted a series research to evaluate the enzymatic-catalyzed degradation of both PET powders and PET films. With the application of ultrasound energy, the biocatalyst was found to be more active and a higher amount of released product was detected, and this activation is more effective on powders than films maybe due to a larger surface area accessible to the enzyme.

Based on the researches above, the possible mechanisms of intensification effect may attribute to a high mass transfer from mechanical effects of ultrasound, which promote a better miscibility of the two reactional initial phases and a better interaction between enzyme and substrates. Another reason relies on an increase of local temperature and pressure, as well as oscillatory fluid motion coming from cavitation effect. The above changes simultaneously promote the interactions of reagents in either homogeneous or heterogeneous system, producing polymers, conjugates or hydrolysate in high efficiency.

## 5 Concluding remarks

The present review enables us to introduce the effect of ultrasound for protein modifications which has seen remarkable development in the last decade. Such ultrasonic processes applied for protein applications have provided more possibilities for various industry areas. The application of ultrasound emulsification on protein emulsion-based products expand significantly their uses as drug delivery systems for intravenous therapeutic and other applications. The sonochemistry could lead to a certain degree changes in secondary and tertiary structures of proteins and endow the proteins with new functionalities. The high mass transfer and the heating effect improve the conversion yield of enzymatic reactions and from products in a short time. Overall, the works presented here demonstrate that ultrasound offers a great potential for protein modifications in food, pharmaceutical and textile industries, which are expected to be promising methodologies for multiple uses.

There is a lack of detailed studies on the effect ultrasound generated radical of the side chain residues of polypeptide chains. Understanding sonochemistry as the effects of those radicals, one can’t expect the loss of protein function by sonochemical effects only unless aromatic residues are located or nearby the active sites of proteins. The enhancement of mass transfer alone by ultrasound will lead to proteins to re-conform their structure leading to the loss the original protein functionality and leading to new support function as shown by multiworks in protein microspheres.

## CRediT authorship contribution statement

**Jing Su:** Conceptualization, Writing - original draft. **Artur Cavaco-Paulo:** Supervision, Conceptualization, Writing - review & editing.

## Declaration of Competing Interest

The authors declare that they have no known competing financial interests or personal relationships that could have appeared to influence the work reported in this paper.
